# Participation of patients with non-psychotic depression in clinical trials: motivating and demotivating factors

**DOI:** 10.1192/j.eurpsy.2026.11655

**Published:** 2026-07-31

**Authors:** N. D. Semenova, L. V. Miller

**Affiliations:** 1 Moscow Research Institute of Psychiatry; 2 Pirogov Russian National Research Medical University, Moscow, Russian Federation

## Abstract

**Introduction:**

Trial recruitment and retention issues are crucial for evidence-based clinical decision-making, as they provide reliable answers to research questions. Quantitative studies often fail to provide an in-depth analysis of the reasons for participant refusal. In contrast, conducting in-depth qualitative studies helps to understand the complex reasons for participants’ refusals, as well as the motivating and demotivating factors that influence patients’ participation in the clinical trials.

**Objectives:**

Our objective was to conduct a qualitative study of patients’ and participants’ views to identify the reasons for refusal (modifiable and unmodifiable), as well as the main motivating and demotivating factors influencing their participation in the study.

**Methods:**

This study examined patients with affective disorders who refused to participate in an ongoing clinical trial (the topic of a state assignment). Semi-structured interviews with five participants (median age 31 years, all men) admitted to the clinic were recorded and transcribed verbatim. Face-to-face qualitative interviews lasted between 30 and 45 minutes and were analysed using thematic analysis.

**Results:**

The results were summarised around key common themes characterising demotivating factors: 1. Lack of therapeutic alliance with the clinician; 2. Lack of personal meaning of the study for the patient; 3. Feeling like an object of influence rather than a subject of interaction. The motivating factor was that patients wanted the study to be responsive to their needs and interests and to have reasonable control over its progress.

**Conclusions:**

Despite the study’s small size and exploratory nature, it offers valuable insights into the factors that motivate and demotivate patients with mood disorders. Although these results may not be generalizable to other groups or women with mood disorders, they offer valuable considerations for future “motivating” interventions in this area (i.e., to address the reason for refusal). In quantitative studies of reasons for rejection, the questions included in the questionnaires are determined by the researchers themselves, and participants are not asked to choose these questions. The scientific and administrative distance of the researchers, as well as their limited understanding of the “living fabric” of the study (situational factors that demotivate the patient), can negatively influence clinical decision-making. Relying on the patient’s perspective facilitates productive interactions between the physician (researcher) and the patient, motivates the patient to participate in the study, and provides more reliable answers to the research questions.

**Disclosure of Interest:**

None Declared

